# Checkrein Deformity of the Hallux and Second Toe after Soft-Tissue Injury

**DOI:** 10.1155/2021/8459586

**Published:** 2021-01-26

**Authors:** J. R. Rodriguez-Collell, D. Mifsut-Miedes

**Affiliations:** ^1^Hospital Malvarrosa de Valencia, Spain; ^2^Universitat de Valencia, Spain

## Abstract

We report the case of a patient who developed a checkrein deformity of the hallux and of the second toe following a direct soft tissue trauma to his right leg, with no associated fracture. This dynamic deformity caused the patient significant trouble walking and prevented him from playing any sport as in the stance phase of gait the toes were forced into maximum plantar flexion and ended up trapped under the foot. An MRI study did not show any bone injury or tendinous tethering. So the cause could be a subclinical compartment syndrome. Treatment consisted of a z-plasty and application of a pulvertaft suture to the flexor hallucis longus. Following a short rehabilitation program (2 weeks), the patient made a full recovery.

## 1. Introduction

Acquired clawing of the toes may be due to contracture of the muscles of the deep posterior compartment of the leg after a subclinic compartment syndrome. This produces a “fixed-length phenomenon” of the long toe flexors, also described as a “checkrein” deformity [1]. The deformity increases with ankle dorsiflexion and decreases with plantar flexion. Another possibility is that the muscle bellies of the tendons have been injured or trapped at the site of the fracture or scarred after a local haematoma. The condition was first described in 1974 by Clawson [2] who observed it following a tibial fracture. Since then, it has been diagnosed following calcaneus fractures, tibial and fibular fractures, removal of a fibular graft, epiphysiolysis of the distal tibia, and, rarely, soft tissue trauma with no fracture [3].

The interest of this study lies in the fact that in our case no fracture was sustained that could account for the deformity, which suggests a subclinical compartment syndrome as an etiology.

This report describes the case of a patient who developed a checkrein deformity of the hallux and of the second digit of the right foot following a direct soft tissue trauma to his right leg, with no associated fracture. Treatment consisted of a z-plasty and application of a pulvertaft suture to the flexor hallucis longus, one double-loop suture at each tendon free-end, and four mattress sutures evenly spaced between. The outcome was satisfactory.

## 2. Case Report

**Figure 1:**
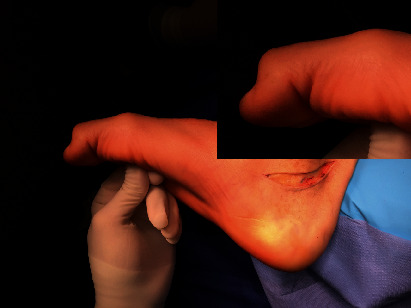
Plantar flexion deformity in the first and second toes of the right foot.

The patient was a 37-year-old male who suffered a car-motorcycle accident that resulted in his right leg being trapped between the bumper of the car and the side of the motorcycle. He was initially diagnosed with a contusion injury on the anteromedial aspect of the right leg. The wound was carefully cleansed and debrided. A year later, the patient presented with a progressive plantar flexion deformity in the first and second toes of the right foot which manifested itself when the ankle was dorsiflexed (Figure 1).

**Figure 2:**
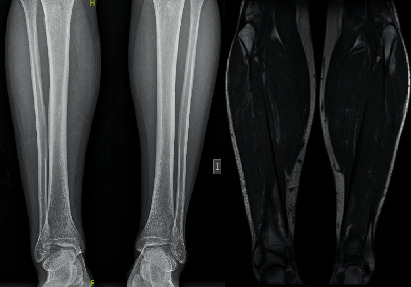
X-ray and MRI study did not show any bony or musculotendinous injury.

This dynamic deformity caused the patient significant trouble walking and prevented him from playing any sport as in the stance phase of gait the toes were forced into maximum plantar flexion and ended up trapped under the foot. Nevertheless, when the ankle was plantar-flexed, active toe flexion and extension was complete. An MRI study did not show any bony or musculotendinous injury (Figure 2). The final diagnosis was a checkrein deformity of the hallux and second toe.

**Figure 3:**
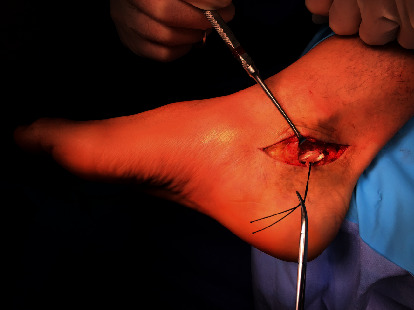
Preliminary suture to keep the hallux in neutral flexion.

The patient was scheduled for surgery. The procedure was performed through a medial retromalleolar approach. After identifying the FHL, it was subjected to a z-tenotomy. A marking stitch was applied to the resulting tendon ends making sure that the great toe remained in neutral flexion/extension while the ankle was also in neutral flexion/extension (Figure 3).

**Figure 4:**
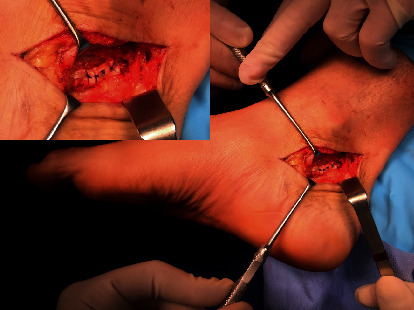
Definitive suture of the flexor hallucis longus type pulvertaft.

Subsequently, a pulvertaft suture was applied (Figure 4). No surgical maneuver was performed on any of the lesser toes.

**Figure 5:**
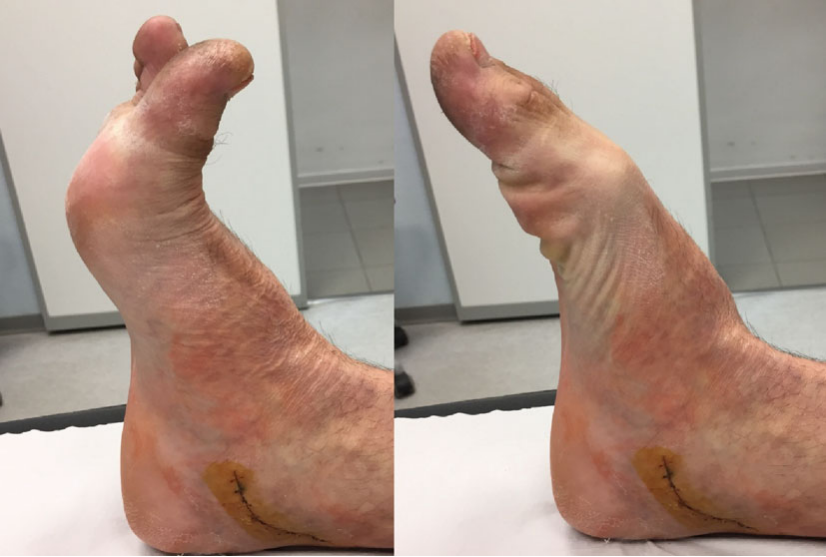
Restoration of active flexion/extension of the great and lesser toes.

After surgery, the patient was immobilized in a splint for 3 weeks, with the ankle in neutral position. Following a short rehabilitation program (2 weeks), the patient made a full recovery, with full restoration of active flexion/extension of the great and lesser toes when the ankle was in neutral dorsiflexion (Figure 5).

## 3. Discussion

Checkrein deformity is a dynamic flexion deformity of the flexor hallucis longus tendon, so that when the ankle is passively dorsiflexed, the metacarpophalangeal and the interphalangeal joint becomes more prominent.

It has been described following tibial fracture, calcaneus fracture, talus, and closed Salter-Harris Type II ankle fracture [1–5]. Lee et al. [3] described 11 cases of checkrein deformity: 10 had a previous fracture and only one case had no fracture but a soft tissue injury. Entrapment of the FHL in the scar tissue or callus at the fracture site, secondary to high-energy trauma, has been associated.

Feeney et al. [1] refer that an explanation of the dynamic deformity could be the development of a subclinical compartment syndrome. An increase in the compartment might result in an injury to the musculotendinous junction of the FHL, with a slight shortening of the tendon due to hypoatrophy and subsequent development of a dynamic deformity.

In our case, there was no entrapment of the tendon but shortening, so we thought that it could be an injury caused by a subclinical compartment syndrome.

The fact that the flexor hallucis longus tendon arises from the lower two-thirds of the posterior aspect of the fibula and the interosseous membrane dictates the kind of surgical technique required to address checkrein deformity. Lee et al. [3] described two types of surgical techniques. The first consists in a release of adhesions and a lengthening of the tendon by a z-plasty at the musculotendinous junction above the ankle at the fracture site; the other technique is based on lengthening the flexor hallucis longus at the midfoot.

Sanhudo and Lompa [4] and Sinnett et al. [5] suggested that lengthening the tendon at the level of the midfoot yields better results than a retromalleolar approach to the ankle. Moreover, as it is sometimes necessary for the lengthening of the FHL and the FDL to be performed separately, an approach to the foot allows the untwining of the connections between the FHL and the FDL. On the other hand, an approach to the fracture site requires a large incision and working in the direction of the neurovascular structures.

However, according to Feeney et al. [1] and Lee et al. [3], lengthening the FHL is sufficient to address a checkrein phenomenon associated with lesser toe deformity given the presence of tendinous interconnections between the FHL and the FDL. The preferred FHL lengthening technique is a z-plasty above the ankle.

In our case, the lengthening procedure was performed at the proximal level, and, although the FDL was spared, the deformity of the second digit was corrected. The reason behind an isolated release of the FHL was that the connections between the FHL and the FDL are located at the level of the distal rearfoot. In our case, there were no tendon adhesions to the tibia, only tendon shortening. At any event, early toe mobilization following injury is crucial, particularly in the presence of a fracture.

## 4. Conclusions

Checkrein deformity is a relatively uncommon condition, which may arise as a result not only of a fracture but also of soft tissue trauma.

An isolated lengthening tenotomy of the FHL at the retromalleolar level is sufficient to correct the associated second-digit deformity.
